# Testing evolutionary models of senescence in a natural population: age and inbreeding effects on fitness components in song sparrows

**DOI:** 10.1098/rspb.2007.0961

**Published:** 2008-01-23

**Authors:** L.F Keller, J.M Reid, P Arcese

**Affiliations:** 1 Zoological Museum, University of ZurichWinterthurerstrasse 190, 8057 Zurich, Switzerland; 2 School of Biological Sciences, Zoology Building, Tillydrone Avenue, University of AberdeenAberdeen AB24 2TZ, UK; 3 Centre for Applied Conservation Research, Department of Forest Sciences, 2424 Main Mall, University of British ColumbiaVancouver, British Columbia, Canada V6T 1Z4

**Keywords:** ageing, gene–environment interactions, life history, longevity, mortality

## Abstract

Mutation accumulation (MA) and antagonistic pleiotropy (AP) have each been hypothesized to explain the evolution of ‘senescence’ or deteriorating fitness in old age. These hypotheses make contrasting predictions concerning age dependence in inbreeding depression in traits that show senescence. Inbreeding depression is predicted to increase with age under MA but not under AP, suggesting one empirical means by which the two can be distinguished. We use pedigree and life-history data from free-living song sparrows (*Melospiza melodia*) to test for additive and interactive effects of age and individual inbreeding coefficient (*f*) on fitness components, and thereby assess the evidence for MA. Annual reproductive success (ARS) and survival (and therefore reproductive value) declined in old age in both sexes, indicating senescence in this short-lived bird. ARS declined with *f* in both sexes and survival declined with *f* in males, indicating inbreeding depression in fitness. We observed a significant age×*f* interaction for male ARS (reflecting increased inbreeding depression as males aged), but not for female ARS or survival in either sex. These analyses therefore provide mixed support for MA. We discuss the strengths and limitations of such analyses and therefore the value of natural pedigreed populations in testing evolutionary models of senescence.

## 1. Introduction

The magnitude of the deleterious effect of inbreeding, also known as inbreeding depression, is a key parameter in evolutionary and population biology (Charlesworth & Charlesworth 1987; Crnokrak & Roff 1999; Hedrick & Kalinowski 2000). However, rather than being constant, the magnitude of inbreeding depression can be state dependent and vary among life-history stages and among individuals experiencing different environmental or genetic conditions (Husband & Schemske 1996; Keller & Waller 2002; Armbruster & Reed 2005; Swindell & Bouzat 2006*a*). Such state dependence in inbreeding depression implies non-independence of non-additive genetic, additive genetic and environmental components of phenotypic variance, and must be understood in order to accurately infer the genetic architecture of phenotypic traits and predict the consequences of inbreeding for genetic variance and population persistence (Falconer & Mackay 1996; Keller & Waller 2002; Swindell & Bouzat 2006*a*).

One state variable of major interest in evolutionary biology is individual age (Charlesworth 1994; Brooks & Kemp 2001). In particular, the observation that fitness often declines in old age, known as ‘senescence’, has attracted considerable attention (Partridge & Barton 1993; Partridge & Mangel 1999). Current evolutionary explanations for senescence comprise two main hypotheses, both derived from the realization that extrinsic mortality reduces the effectiveness of natural selection against late-acting deleterious alleles (Partridge & Barton 1993; Charlesworth 1994; Partridge & Mangel 1999). ‘Mutation accumulation’ (MA) proposes that senescence reflects the higher frequency of late-acting deleterious alleles expected under mutation-selection balance, while ‘antagonistic pleiotropy’ (AP) proposes that senescence results from directional selection for alleles with beneficial early effects but deleterious late effects (Medawar 1952; Williams 1957; Partridge & Barton 1993). While these hypotheses are not mutually exclusive, it is valuable to assess the evidence for each, and therefore their relative roles in causing senescence, in order to predict the probable phenotypic and genetic consequences of environmentally or experimentally induced changes in life history (Partridge & Barton 1993; Charlesworth & Hughes 1996; Hughes *et al*. 2002).

Population genetic theory makes contrasting predictions concerning the degree of age dependence in inbreeding depression expected under MA and AP. Specifically, inbreeding depression in phenotypic traits that show senescence is predicted to be greater at older ages under MA but not under AP (Charlesworth & Hughes 1996). These contrasting predictions, which explicitly concern the degree of state dependence in inbreeding depression, provide one valuable empirical means of distinguishing MA and AP (Partridge & Barton 1993; Charlesworth & Hughes 1996). Studies of age dependence in inbreeding depression therefore have the potential to provide insights into the genetic architecture of senescence, although observed patterns must be interpreted with due regard to other processes that may cause variation in the magnitude of inbreeding depression.

Recent studies have used laboratory populations of *Drosophila melanogaster*, *Callosobruchus maculatus*, *Callosobruchus chinensis* and *Stator limbatus* to test whether inbreeding depression in fitness components increases with age, as predicted under MA (Tanaka 1990; Charlesworth & Hughes 1996; Hughes *et al*. 2002; Snoke & Promislow 2003; Fox *et al*. 2006; Swindell & Bouzat 2006*b*). However, such experiments often use inbred lines maintained under laboratory regimes that may select for early fecundity and artificially increase the load due to late-acting deleterious mutations (Partridge & Barton 1993; Promislow & Tatar 1998). Studies of captive populations may also fail to detect inbreeding depression and gene–environment interactions arising in natural environments (Crnokrak & Roff 1999; Joron & Brakefield 2003). It is therefore valuable to replicate laboratory studies in natural populations (Snoke & Promislow 2003). However, the prediction that inbreeding depression in senescing fitness traits increases with age under MA has rarely been tested in natural populations, probably because such analyses require substantial pedigree and life-history information.

We used a natural song sparrow (*Melospiza melodia*) population, for which unusually comprehensive pedigree data exist, to quantify additive and interactive effects of age and inbreeding on major fitness components. First, we test whether annual reproductive success (ARS) and adult survival (and therefore reproductive value) decline in old age, and thereby assess the evidence for senescence in this relatively short-lived bird. Second, we test whether ARS and survival also show inbreeding depression. Finally, we test for age×inbreeding interactions to determine whether inbreeding depression increased in old age, as predicted under MA but not under AP. We discuss the strengths and limitations of these analyses and therefore the value of natural pedigreed populations in testing evolutionary models of senescence.

## 2. Material and methods

### (a) Study population

Mandarte Island, approximately 6 ha in size, lies 25 km north east of Victoria, British Columbia, Canada. Its resident population (*n*=4–74 breeding pairs) of song sparrows (*M. melodia*) has been studied intensively since 1975. Full details of the study population and field methods are reported by Smith *et al*. (2006). Briefly, each year except 1980 when fieldwork was less intensive, all breeding attempts were monitored and all fledglings were individually colour ringed before leaving their natal territory. Adults typically breed one to three times per year (range 0–6 years) starting from age 1. Immigrants to the breeding population (1.1 yr^−1^ on average) could be distinguished from existing natives because they had been individually colour ringed as chicks elsewhere, or because they were unringed upon arrival on Mandarte. All unringed immigrants were caught and individually colour ringed soon after settling. Since dispersal primarily occurs prior to first breeding, these few immigrants of unknown origin were assumed to be of age 1 in their first spring on Mandarte. During each spring, the identities of surviving adults and survival of colour-ringed fledglings to independence from parental care (approx. 12 days post-fledging) were recorded, allowing adult survival probabilities and ARS (the total number of offspring raised to independence) to be estimated. Analyses of age-specific variation in ARS and survival initially included all adults alive on Mandarte during 1975–2006, except that individuals involved in supplementary feeding experiments in 1979, 1985 and 1988 were excluded.

We used long-term data on social pairings to compile a complete pedigree for the population, covering all sparrows hatched since 1981 with some lineages extending to 1975 (Keller 1998; Reid *et al*. 2007). We used standard algorithms to estimate each individual's coefficient of inbreeding (*f*) and the coefficient of kinship between mates directly from the pedigree (Falconer & Mackay 1996). While immigrants to Mandarte are of unknown *f*, they are genetically distinguishable from Mandarte natives at the time of arrival (Keller *et al*. 2001; Reid *et al*. 2006). Offspring of immigrant–native pairings can therefore be defined as outbred (*f*=0; Marr *et al*. 2002). Extra-pair fertilizations (EPFs) occur on Mandarte (approx. 25% of offspring hatched during 1993–1996; O'Connor *et al*. 2006), introducing error into the pedigree and estimates of individual *f*. However, EPFs were not more frequent in females that were more closely related to their social mates, and females were no more or less closely related to their extra-pair mate than to their social mate (see Reid *et al*. 2007). EPFs are therefore likely to introduce error but not bias into estimates of individual *f*, meaning that the magnitude of inbreeding depression in phenotypic traits is likely to be underestimated (Keller *et al*. 2002; Kruuk *et al*. 2002; Marr *et al*. in preparation).

We used generalized linear mixed models with negative binomial errors to test whether ARS varied with age and/or *f* (Proc Glimmix, SAS Institute; Keller *et al*. 2006). Since reproductive success is a trait of a pair of breeders, we tested for the effects of individual *f*, mate *f* and kinship (*k*) between them. Models included two random factors: year (since ARS varies among years; Smith *et al*. 2006) and individual identity to account for non-independence among multiple observations of long-lived individuals. Since the annual probability of resighting a living song sparrow on Mandarte is effectively one, we used proportional hazards models for interval-censored data to test whether the probability of adult survival from one spring to the next (hereafter termed ‘survival’) varied with age and/or *f* (Heisey 1992; Keller 1998). Models were stratified by hatch year, with age and *f* fitted as covariates (thereby allowing comparison of survival among individuals of differing age and *f*, which hatched simultaneously). Observations of individuals alive in 2006 were right censored. Analyses were restricted to individuals that survived to adulthood (age 1) since apparent survival from fledging to age 1 includes unknown rates of natal dispersal and is not directly comparable to estimated survival through subsequent years. We did not fit Gompertz models, commonly used to measure age dependence in survival (Snoke & Promislow 2003; Swindell & Bouzat 2006*b*), because data were collected in discrete annual intervals rather than continuously. However, exploratory analyses suggested that exponential rates of age-dependent mortality estimated by Gompertz models were significantly positive, in agreement with reported proportional hazards models.

Apparent age-specific variation in life histories observed across populations can reflect heterogeneities in life history among population members rather than age-specific variation occurring in individuals. For example, average ARS could increase across initial age classes because poor breeders die early (Nol & Smith 1987; Forslund & Pärt 1995), and may subsequently decline if individuals that reproduce at a low rate survive longer (Reid *et al*. 2003). In practice, effects of among-individual variation in reproductive success may be small relative to within-individual variation (Newton & Rothery 2002; Velando *et al*. 2006; Low *et al*. 2007). However, to verify this assumption, we repeated analyses of ARS across a restricted dataset comprising individuals that survived 4–6 years in total. In the case of survival, it is harder to envisage how artefactual declines with age could arise. Variation in excess risk (‘frailty’) among individuals can obscure evidence of declining survival in old age since individuals surviving longest typically have low frailties (McDonald *et al*. 1996; Service 2000), but an artefactual decline could only arise if individuals with high frailties survive longest. Declining average survival with age is therefore likely to reflect declining survival in individuals. Finally, within-individual declines in phenotype with age may reflect state-dependent reallocation of resources to other traits rather than senescence *per se*. Therefore, to examine the evidence for true senescence in fitness rigorously; we calculated age-specific reproductive values. Reproductive values are fundamental quantities that combine survival and fecundity into a single integrated value that represents an age class' relative contribution to the future population (Charlesworth 1994; Newton & Rothery 1997). Age-specific reproductive values were calculated for each cohort separately following Newton & Rothery (1997), and age-specific averages are presented. Since values are highly interdependent within each cohort, estimates of reproductive value were not themselves subject to statistical analysis but were used to substantiate patterns of age-specific variation observed in ARS and survival.

Since estimates of *f* are sensitive to pedigree depth (Falconer & Mackay 1996), analyses including *f* were restricted to individuals where all four grandparents were known and/or where at least one parent was an immigrant (where offspring *f*=0, see above). Results remained quantitatively similar if analyses were restricted to individuals hatched since 1990 (for which the pedigree depth is substantial; Marr *et al*. 2006; Reid *et al*. 2006). Age was modelled as linear and quadratic variables, reflecting expected linear or ‘domed’ relationships between age and fitness components (e.g. Newton & Rothery 1997; Reid *et al*. 2003; Low *et al*. 2007). Where preliminary analyses indicated quadratic effects, observed ages (*a*_obs_) were centred by subtracting the mean observed age (thus *a*_c_=*a*_obs_−*ā*_obs_), thereby reducing multicollinearity between *a*_obs_ and $a_{\text{obs}}^{2}$ from approximately 0.95 to approximately 0.70 (Draper & Smith 1981). Since quadratic models can be significant when traits reach asymptotes rather than peaks, we used the same mixed and proportional hazards models to test explicitly whether traits declined significantly with age when only data after the empirical peaks were included. Exploratory analyses provided no evidence of quadratic effects of *f*. Non-significant main effects and interactions were eliminated from models sequentially, and associated statistics estimated by reintroduction to final models. Data from males and females were analysed separately except where analyses were designed to explicitly test for between-sex differences. Results remained quantitatively similar when immigrants were excluded. Analyses were run in SAS (v. 9.1.3, SAS Institute, Inc.). Means are presented ±1 s.d. For graphical presentation and to determine the functional form of the relationships (but not for hypothesis testing), age-specific ARS estimates were standardized for among-year variation by calculating *z*-scores, and survival estimates were plotted as Martingale residuals of a null model adjusting for hatch year (Therneau & Grambsch 2000, p. 91). Estimated reproductive values were not corrected for among-year variation.

## 3. Results

### (a) Age effects

**Figure 1:**
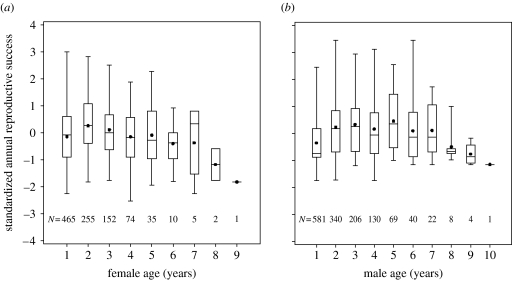
Age-specific variation in (year-standardized) ARS in (*a*) female and (*b*) male song sparrows. Boxes, 25th, 50th (median) and 75th percentiles; dots, the means; lines, the full range of values. Sample sizes for each age class are listed below the boxes.

Across breeding song sparrows, female age averaged 2.0±1.3 years (median=2 years, range 1–9 years) and male age averaged 2.3±1.6 years (median=2 years, range 1–10 years). ARS declined in old age in both sexes (figure 1; table 1). The declines from peak ARS at age 2 (females) and age 5 (males) were statistically significant (*p*=0.016 and 0.023, respectively). Final models and parameter estimates were similar when analyses were restricted to individuals that survived 4–6 years (table 1), suggesting that population-wide variation in ARS primarily reflected longitudinal variation in individuals.

**Figure 2:**
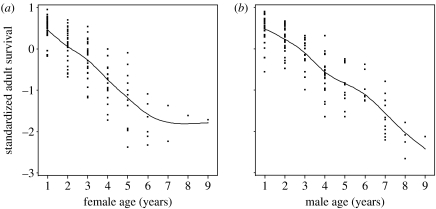
Age-specific variation in (year-standardized) annual adult survival in (*a*) female and (*b*) male song sparrows. Martingale residuals of a null model adjusting for hatch year are shown, smoothed using a cubic spline.

**Figure 3:**
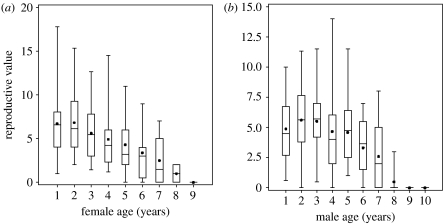
Age-specific variation in reproductive value in (*a*) female and (*b*) male song sparrows. Values were calculated separately for each of 30 cohorts. Boxes, 25th, 50th (median) and 75th percentiles; dots, the means; lines, the full range of values.

Survival declined markedly with age in males and females, with no clear evidence of nonlinear effects (figure 2; table 2). The apparent levelling off in hazard in old females was not significant (*p*>0.44) and reflects a very small sample size (*n*=4) of females aged 7 or more years. Average reproductive values declined markedly in old age in both sexes (figure 3).

### (b) Inbreeding effects

Female *f* averaged 0.034±0.053 (range 0.000–0.305) and male *f* averaged 0.032±0.052 (range 0.000–0.305). ARS declined markedly with increasing individual *f* in both females and males (table 1). However, the age×*f* interaction was significant only among males (table 1). ARS also declined with mate *f* in females (and showed a non-significant trend in males), but did not vary significantly with *k* (see also Keller 1998). Final models and parameter estimates were quantitatively similar when analyses were restricted to individuals that survived 4–6 years.

Survival declined with increasing *f* in males but not females (table 2). Analysis of combined data from both sexes revealed a significant sex×*f* interaction (*p*=0.018; see also Keller *et al*. 2006). However, age×*f* interactions were not significant in either sex (table 2). All results remained qualitatively similar even when the most inbred individuals were excluded from the analyses.

## 4. Discussion

Precise knowledge of age-specific variation in fitness is critical to understanding life-history evolution and dynamics of age-structured populations (Charlesworth 1994; Coulson *et al*. 2001; Altwegg *et al*. 2007), and is therefore the focus of considerable research (e.g. Forslund & Pärt 1995; Komdeur 1996; Newton & Rothery 1997; Nichols *et al*. 1997; Loison *et al*. 1999; Møller & de Lope 1999; McElligott *et al*. 2002; Reid *et al*. 2003; Velando *et al*. 2006; Low *et al*. 2007). The slight increase in mean ARS across younger age classes in male song sparrows may reflect early mortality of poor breeders, initial reproductive restraint and/or increasing learning, experience or social status (see Nol & Smith 1987; Forslund & Pärt 1995; Robertson & Rendell 2001; Low *et al*. 2007 and references therein). However, of primary current interest, ARS, survival and reproductive value all declined in old age in both sexes (figures 1–3). These declines are unlikely to be artefacts of heterogeneity in individual life histories because similar patterns were evident when analyses were restricted to relatively long-lived individuals, and analyses controlled for temporal variation in environment. Thus, these data strongly indicate that senescence occurs in song sparrows. Recent studies of relatively short-lived birds have reported age-specific declines in physiology (Lavoie 2006), reproductive success (e.g. Komdeur 1996; Robertson & Rendell 2001; Low *et al*. 2007) and survival (e.g. McDonald *et al*. 1996; Orell & Belda 2002, but see Nichols *et al*. 1997; Altwegg *et al*. 2007). However, few studies have demonstrated declines in both ARS and survival, and therefore provided compelling evidence of senescence as opposed to state-dependent reallocation of resources among life-history components (but see Newton & Rothery 1997; Møller & de Lope 1999; Reid *et al*. 2003).

Consistent with previous analyses of smaller datasets, ARS declined with *f* in both sexes and survival declined markedly with *f* in males but not females (see Keller 1998; Keller *et al*. 2006). While estimates of inbreeding depression in reproductive success are increasingly common, inbreeding effects on adult survival have rarely been quantified in free-living animals, perhaps because inbreeding depression in early survival can be severe enough to eliminate all inbred individuals (Keller & Waller 2002; Keller *et al*. 2002; Kruuk *et al*. 2002). Current analyses suggest that inbreeding depression in adult survival can be substantial, but also sex specific (see Fox *et al*. (2006), Keller *et al*. (2006) and Reid *et al*. (2007) for discussion of sex-specific inbreeding depression). In summary, substantial inbreeding depression was evident in major fitness components in Mandarte's song sparrows.

We observed a significant age×*f* interaction in male ARS, indicating increased inbreeding depression in older males. This pattern is consistent with the mutation accumulation hypothesis of senescence (Charlesworth & Hughes 1996) and adds evidence from free-living song sparrows to existing support for MA, derived largely from studies on *D. melanogaster* (Charlesworth & Hughes 1996; Hughes *et al*. 2002; Snoke & Promislow 2003; Swindell & Bouzat 2006*b*). However, since the magnitude of inbreeding depression can increase with environmental severity (Armbruster & Reed 2005), it is also possible that age×*f* interactions could arise in the absence of MA as a consequence of pleiotropic effects on homeostasis or an individual's ability to acquire resources in old age (Charlesworth & Hughes 1996; Fox *et al*. 2006). Moreover, we detected no age dependence in inbreeding depression in female ARS or survival in either sex, and therefore found no evidence for MA with respect to these traits. Since the causes of inbreeding depression in ARS differ between males and females (inbred males make fewer nesting attempts while inbred females have lower hatching success; Keller 1998; Keller *et al*. 2006), the apparent age×*f* interaction in males but not in females may indicate that mutational loads differ between the life-history components that underlie inbreeding depression in ARS in each sex.

Some laboratory studies have also failed to find age×*f* interactions and have consequently suggested an absence of MA (Tanaka 1990; Fox *et al*. 2006). In our analyses, however, the large CIs surrounding some parameter estimates (tables 1 and 2) indicate that our statistical power to detect age×*f* interactions may have been low. For example, any underestimation of inbreeding depression due to EPFs, or of the rate of senescence due to individual variation in frailty, would probably reduce power. Nevertheless, our null results are unlikely to solely reflect a lack of statistical power because point estimates for interactions were small and indicated reduced inbreeding depression in old age for survival (opposite to the change predicted under MA). However, it remains possible that biases in mortality could obscure increases in inbreeding depression with age in cross-sectional analyses. For example, the inbred sparrows that survived to relatively old age may have been particularly viable as a consequence of chance variation in mutational loads within estimated values of *f*. While laboratory studies are not immune to such problems (Promislow & Tatar 1998; Hughes *et al*. 2002; Fox *et al*. 2006), selection may be more severe in the wild, making analyses of free-living populations more susceptible to this problem. Overall, therefore, our results provide equivocal support for MA. We suggest that evidence from single natural populations be interpreted with caution and that further empirical tests of evolutionary models of senescence in natural populations are required before any general picture emerges (see also Charmantier *et al*. 2006).

In conclusion, analyses of comprehensive pedigree and life-history data from song sparrows revealed age dependence in inbreeding depression, which was consistent with MA, in one of four sex-specific fitness components. While an absence of age-dependent inbreeding depression is consistent with AP, a failure to invalidate a null expectation does not provide strong support for any hypothesis. Nevertheless, our results are broadly consistent with the current consensus emerging from experiments on *D. melanogaster* that AP may be the primary cause of senescence with MA probably contributing further (Hughes & Reynolds 2005). A valuable future objective would be to test whether dominance variance, or the ratio of dominance to additive genetic variance, varies with age in phenotypic traits that show senescence, as predicted under MA (Charlesworth & Hughes 1996; Snoke & Promislow 2003). Such analyses will pose major challenges to even the best natural pedigreed population.

## Figures and Tables

**Table 1:** Models relating ARS to age, inbreeding coefficients (*f*) and kinship coefficient (*k*) in female and male song sparrows. (*F* and *R* indicate the full dataset and restricted dataset of individuals that lived 4–6 years, respectively. *N*_ind_ and *N*_obs_ are the sample sizes of individuals and observations, respectively. Individual *f* and mate *f* are the inbreeding coefficients of focal individuals and their mates, respectively. Sample sizes were reduced in analyses including *f* because individuals with unknown parents or grandparents were excluded. ‘Individual’ and ‘year’ represent the estimates of the variance components associated with the two random effects.)

dataset	*N*_ind_	*N*_obs_	age		age^2^		individual *f*		mate *f*		kinship (*k*)		age×individual *f*		year	individual
							
			*F*(*p*)	*β*(95% CI)	*F*(*p*)	*β*(95% CI)	*F*(*p*)	*β*(95% CI)	*F*(*p*)	*β*(95% CI)	*F*(*p*)	*β*(95% CI)	*F*(*p*)	*β*(95% CI)	estimate±s.e.	estimate±s.e.
*females*																
*F*	452	999	11.8	0.09	22.4	−0.05									0.19±0.06	0.07±0.02
			(0.0006)	(0.04 to −0.13)	(<0.0001)	(−0.07 to −0.03)										
*R*	84	340	0.3	0.03	1.5	−0.03									0.18±0.06	0.05±0.02
			(0.59)	(−0.07 to 0.12)	(0.22)	(−0.07 to 0.02)										
*F*	325	717	6.97	0.09	13.3	−0.04	7.03	−1.52	5.99	−1.24	1.08	0.46	0.01	−0.04	0.19±0.06	0.07±0.02
			(0.009)	(0.02 to 0.16)	(0.0003)	(−0.06 to −0.02)	(0.008)	(−2.64 to −0.40)	(0.015)	(−2.23 to −0.24)	(0.30)	(−0.41 to 1.33)	(0.93)	(−0.80 to 0.73)		
*males*																
*F*	579	1401	139.4	0.31	96.2	−0.09									0.33±0.09	0.15±0.03
			(<0.0001)	(0.26 to 0.37)	(<0.0001)	(−0.11 to −0.07)										
*R*	122	513	23.7	0.20	8.4	−0.06									0.18±0.06	0.12±0.04
			(<0.0001)	(0.12 to 0.28)	(0.004)	(−0.09 to −0.02)										
*F*	327	912	45.02	0.23	41.3	−0.07	4.22	−1.38	3.1	−1.18	1.67	−0.78	4.24	−0.87		
			(<0.0001)	(0.16 to 0.30)	(<0.0001)	(−0.09 to −0.05)	(0.04)	(−2.71 to −0.06)	(0.08)	(−2.50 to 0.14)	(0.20)	(−1.96 to 0.40)	(0.04)	(−1.70 to −0.04)	0.29±0.09	0.09±0.03

**Table 2:** Proportional hazards models relating variation in the annual probability of adult survival to age and inbreeding coefficient (*f*) in female and male song sparrows. (Positive and negative parameter estimates indicate increased and reduced hazard functions, and therefore increased and reduced probabilities of death, respectively. *N*_obs_ and *N*_mort_ are the sample sizes of observations and mortality events, respectively. Sample sizes were reduced in analyses including *f* because individuals with unknown grandparents were excluded.)

*N*_obs_	*N*_mort_	age		*f*		age×*f*		year of birth
			
		*Χ*^2^ (*p*)	*β* (95% CI)	*Χ*^2^ (*p*)	*β* (95% CI)	*Χ*^2^ (*p*)	*β* (95% CI)	*Χ*^2^ (*p*)
*females*								
819	311	9.1 (0.003)	0.14 (0.05 to 0.23)					46.4 (0.008)
640	249	8.0 (0.005)	0.15 (0.05 to 0.24)	0.8 (0.38)	−1.13 (−3.70 to 1.44)	0.1 (0.83)	−0.20 (−2.05 to 1.64)	42.8 (0.010)
*males*								
1309	470	12.8 (<0.0001)	0.11 (0.05 to 0.17)					64.5 (<0.0001)
1031	365	13.3 (0.0003)	0.13 (0.06 to 0.20)	7.7 (0.006)	2.82 (0.91 to 4.74)	0.3 (0.58)	−0.33 (−1.49 to 0.83)	50.1 (0.002)
